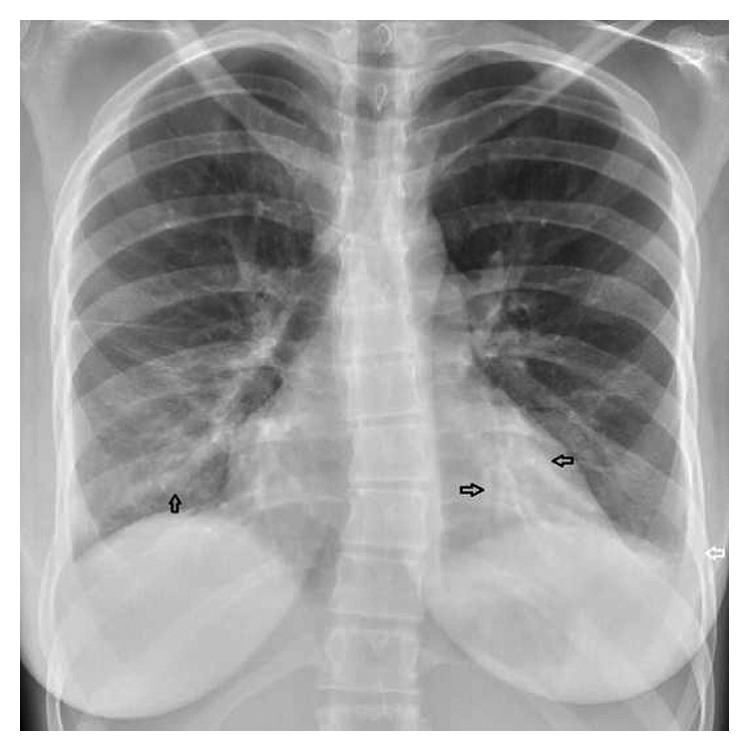# Pulmonary Systemic Lupus Erythematosus Mimicking a Pneumonia in a Postpartum Female

**DOI:** 10.1155/2018/5379192

**Published:** 2018-07-24

**Authors:** Varun Jain, Maryann Aziz, Mina G. Banoub, Jeremy T. Neuman, Richard Sidlow

**Affiliations:** ^1^Department of Pediatrics, Staten Island University Hospital-Northwell Health, Staten Island, NY, USA; ^2^Department of Internal Medicine, St. Joseph's Regional Medical Center, Paterson, NJ, USA; ^3^Division of Pediatric Radiology, Department of Radiology, Staten Island University Hospital-Northwell Health, Staten Island, NY, USA; ^4^Division of Pediatric Hospitalist Medicine, Department of Pediatrics, Staten Island University Hospital-Northwell Health, Staten Island, NY, USA

## Abstract

The pulmonary manifestations of systemic lupus erythematosus can range in severity from mild to life threatening and can be particularly marked in women who are recently postpartum. We present below a seventeen-year-old female patient, one month postpartum, who had findings consistent with an acute infectious pneumonia whom upon further query and passage of time was diagnosed with severe pneumonitis due to systemic lupus erythematosus.

## 1. Introduction

Pulmonary systemic lupus erythematosus (SLE) is usually a late manifestation of the disease. However, patients with latent SLE may present with pulmonary symptoms without meeting formal criteria for diagnosis [1]. In addition, abnormal pulmonary function tests and/or chest radiographs may be detected in rheumatologically asymptomatic patients [2]. The clinical presentation of rheumatologic lung disease can involve all anatomic and histologic components of the respiratory system whose symptoms include cough with or without phlegm production, hemoptysis, dyspnea, pleuritic pain, or hypoxemia, with lung involvement ultimately being either unilateral or bilateral [3]. Pleuritis is the most common pulmonary complaint in adult patients (45–60% of cases) and is detected in 50–83% of autopsies of lupus patients [4, 5]. In children diagnosed with SLE, pulmonary involvement is common, could be potentially life threatening, and may even herald the onset of the disease, with the most common pulmonary symptom in this age group being chest pain [6–8].

Acute lupus pneumonitis can closely mimic an acute infectious pneumonia both clinically and radiographically. Clinically, both present with dyspnea, fever, and chest pain. Radiographically, SLE pneumonitis and infectious pneumonitis both present with patchy areas of consolidation, traction atelectasis, honeycomb changes, or pleural effusions [9].

During pregnancy or soon after delivery, women with previously diagnosed SLE are at higher risk of preeclampsia, miscarriage, intrauterine growth retardation of their fetus, preterm delivery, in addition to exacerbation of their underlying condition, worsening renal impairment and hypertension, and venous thromboembolism [10, 11].

We report below on a seventeen-year-old African American female, one month postpartum, who complained of pleuritic chest pain and cough for two weeks. A chest X-ray revealed evidence of right basilar opacity and bilateral pleural effusions, initially diagnosed as pneumonia but subsequently found to be part of an initial presentation of SLE.

## 2. Case Report

A 17-year-old African American female with a negative past medical history except intermittent asthma presented to the emergency room complaining of cough and chest pain for the past two weeks in addition to rib pain, back pain, and weakness for about one month. The chest pain had been worsening upon deep inspiration over the past few days. The pain was unlike what the patient had experienced previously associated with acute asthma exacerbations and was refractory to beta-agonist treatment.

One month prior, she had given birth to a 32-week gestation infant after an unremarkable pregnancy and delivery. Two weeks prior to this presentation, the patient had been seen in another emergency room for similar symptoms. A chest X-ray and computed tomography (CT) of the chest was performed then which were read as normal.

Upon examination in the emergency room, the patient was noted to be afebrile and on pulse oximetry was saturating at 98% on room air. An electrocardiogram showed sinus tachycardia at 110 beats per minute with possible left atrial enlargement and no S-T segment or T-wave abnormalities. A chest X-ray revealed a right basilar opacity and bilateral pleural effusion consistent with a diagnosis of pneumonia (Figure 1). Treatment with intravenous antibiotics was initiated, and the patient was transferred to the pediatric floor.

On the floor, additional history of having experienced swelling of her lower limbs and joint pain in her hands was obtained. Physical examination revealed the following findings: right-sided metacarpophalangeal, wrist, elbow, and knee swelling and erythema, decreased range of motion of both knees, bilateral conjunctival erythema, bilateral nonpitting pedal edema, mild diffuse abdominal tenderness, a confluent erythematous maculopapular rash involving both upper and lower extremities, and a malar rash.

Laboratory investigations were notable for a white blood cell count of 2.4 thousand per cubic millimeter (nl 4.8–10.8 thousand per cubic millimeter) with 84% neutrophils (nl 40–80%), 9% bands (nl 0–6%), and 6% lymphocytes (nl 15–50%), a creatinine level of 1.24 milligrams per deciliter (nl 0.3–0.8 milligrams per deciliter), hemoglobin level of 9.6 grams per deciliter (nl 10.7–17.3 grams per deciliter), an albumin level of 2.9 grams per deciliter (nl 2.7–4.8 grams per deciliter), an erythrocyte sedimentation rate of 72 millimeters per hour (nl 0–20 millimeters per hour), antinuclear antibody titer of 1:2560 with a homogeneous pattern (nl < 1 :80), and an anti-double-stranded DNA antibody level of >1000 iU/ml (nl ≤ 30). Urinalysis was positive for protein of 100 milligrams per deciliter (nl negative) and white blood cells of 12–20 per high power field (nl negative). This was followed by a spot urine protein-to-creatinine ratio which revealed significant proteinuria (871 millligrams per gram creatinine (nl 80–200 milligrams per gram creatinine)) consistent with SLE related kidney disease. All other laboratory values including rheumatoid factor, complement studies, and coagulation studies were normal. Additionally, bilateral lower extremity venous duplex studies showed no evidence of deep vein thromboses.

After approximately forty-eight hours on the pediatric floor, the diagnosis of SLE was confirmed—the antibiotics were discontinued and the patient was started on high-dose intravenous methylprednisolone. Soon after the steroids were started, the patient became hypertensive (180/114 mmHg) and bradycardic with unbearable headaches prompting transfer to the pediatric intensive care unit. Upon transfer, the patient's physical and neurologic examinations were normal except for mildly decreased air entry at the lung bases. An echocardiogram was performed which showed an ejection fraction of 55–60%, mild to moderate mitral regurgitation, peak systolic pulmonary artery pressure of 45 mmHg (moderately increased), normal-sized pulmonary arteries, moderate to severe tricuspid regurgitation, a mildly dilated right atrium, and a less than 50% variation in respirophasic changes in the inferior vena cava and hepatic veins. The patient was started on intravenous hydralazine and oral nifedipine. A second chest X-ray was unchanged. A repeat electrocardiogram showed marked sinus bradycardia at 42 beats per minute with a prolonged QTc interval of 462 milliseconds and flipped T waves in the anterior leads. The patient was then transferred to a tertiary facility for management of her pulmonary hypertension, dysrhythmia, and further investigation and management of her nephrologic and rheumatologic disease.

## 3. Discussion

Several cases of lupus pneumonitis mimicking pneumonia are reported in the pediatric medical literature [12–14]. One case involves bronchiolitis obliterans organizing pneumonia in a 16-year-old patient not yet diagnosed with SLE. The patient presented with dyspnea, cough, arthralgia, malar rash, and fine crackles heard on lung exam. After no response to antibiotics, a rheumatologic workup was performed revealing positive results for dsDNA and ANA tests: steroid treatment was initiated followed with improvement in the patient's symptoms [15]. In another case, acute lupus pneumonitis was found to mimic infective pneumonia, also not responding to antibiotics and improving with corticosteroids [12]. Another case involved a 19-year-old female who presented with intermittent high-grade fevers, chills, and an erythematous rash all over her body. Radiographically, the patient had a pneumonia with a pleural effusion which quickly progressed to what was diagnosed as fulminant lupus pneumonitis requiring mechanical ventilatory support [13]. In all cases, the classic symptoms of lupus were present to help steer the diagnosis towards SLE, symptoms such as malar rash, pancytopenia, and arthralgias. It is possible in our case that the immune downregulation associated with pregnancy delayed the crudescence of rheumatologic symptoms or attenuated preexisting milder symptoms, only to return postpartum in a more florid and severe form. Diagnostic anchoring on her pulmonary symptoms, confirmed with positive chest X-ray findings that mimic infectious pneumonia closely, contributed to the delay in obtaining our patient's ultimate diagnosis.

A rare complication and poor prognostic factor of lupus pneumonitis is pulmonary hypertension (PH), a complication our patient experienced. Histologically speaking, it is most commonly due to plexiform angiomatous lesions in the arterial media that thicken the arterial wall and concomitantly reduce its compliance. Defined in its most severe form as a pulmonary systolic pressure estimated by echocardiography greater than 40 mmHg, it occurs in one percent of SLE patients [16]. In conjunction with lupus nephritis, SLE with PH confers greater mortality risk, a combination relevant to our patient above [17, 18].

## 4. Conclusion

Our patient's diagnostic course presents a cautionary tale with several key points. Firstly, all radiographic pneumonias are not infectious in nature, and when antibiotic therapy is proving ineffectual in a patient, a rheumatologic cause for the patient's findings must be considered. Secondly, pulmonary SLE can present in a dramatic, rapidly worsening fashion, especially in a postpartum female. Lastly, when a patient is diagnosed with SLE, involvement of the pulmonary system must be ruled out, even when asymptomatic from a pulmonary standpoint.

## Figures and Tables

**Figure 1:**